# Development of Prediction Model to Estimate the Risk of Heart Failure in Diabetes Mellitus

**DOI:** 10.3389/fcvm.2022.900267

**Published:** 2022-07-01

**Authors:** Hongling Qu, Cuiyun Wu, Peiji Ye, Weibiao Lv

**Affiliations:** ^1^Department of Clinical Laboratory, Shunde Hospital, Southern Medical University (The First People’s Hospital of Shunde), Foshan, China; ^2^Department of Blood Transfusion, Shunde Hospital, Southern Medical University (The First People’s Hospital of Shunde), Foshan, China

**Keywords:** prediction, nomogram, risk factors, diabetes mellitus, heart failure

## Abstract

**Background:**

Heart failure (HF) is a leading cause of mortality and disability in patients with diabetes mellitus (DM). The aim of the study is to predict the risk of HF incidence in patients with DM by developing a risk prediction model.

**Methods:**

We constructed a regression model based on 270 inpatients with DM between February 2018 and January 2019. Binary logistic regression was applied to develop the final model incorporating the predictors selected by least absolute shrinkage and selection operator regression. The nomogram was estimated with an area under the receiver operator characteristic curve and calibration diagram and validated with the bootstrap method.

**Results:**

Risk factors including age, coronary heart disease (CHD), high-density lipoprotein (HDL), and low-density lipoprotein (LDL) were incorporated in the final model as predictors. Age ≥ 61 years old, LDL, and CHD were risk factors for DM with HF, with odds ratios (ORs) of 32.84 (95% CI: 6.74, 253.99), 1.33 (95% CI: 1.06, 1.72), and 3.94 (95% CI: 1.43, 13.43), respectively. HDL was a protective factor with an OR of 0.11 (95% CI: 0.04, 0.28). The area under curve of the model was 0.863 (95% confidence interval, 0.812∼0.913). The plot of the calibration showed that there was a good consistency between predicted probability and actual probability. Harrell’s C-index of the nomogram was 0.845, and the model showed satisfactory calibration in the internal validation cohort.

**Conclusion:**

The prediction nomogram we developed can estimate the possibility of HF in patients with DM according the predictor items.

## Introduction

The incidence and mortality rate of diabetes mellitus (DM) are increasing in recent years, especially in developing countries (1, 2). Cardiovascular disease (CVD) is a major complication of blood glucose dysregulation (3). Patients with diabetes have a 2- to 4-fold increased risk of heart failure (HF) compared with those without diabetes (4). There is higher prevalence, incidence, and mortality in diabetic patients with HF compared with those with diabetes who remain HF-free (5–7). In population-based studies, concomitant DM increases the risk of death in both hospitalized and ambulatory patients with HF (8–11). It is important to note that even in patients with prediabetes, the risk of HF is increased and associated with poor prognosis (12, 13).

DM and HF have considerable morbidity and mortality, when they occur together, which further worsens adverse patient outcomes, quality of life, and costs of care (4). It is important to find the risk factors of diabetes complicated by HF. Therefore, we aimed to develop a simplified prediction model to identify the high-risk of HF early in diabetic patients and conduct early intervention for them.

## Materials and Methods

### Patients

We conducted a trial on a population of resident patients diagnosed with DM in Southern Medical University (The First People’s Hospital of Shunde) between February 2018 and January 2019. In total, 270 patients were included. Our outcome was diabetes mellitus with heart failure (DM-HF) disease. We excluded patients who had suffered from DM-HF and received treatment. DM was defined as use of medications for diabetes or fasting blood glucose ≥ 7 mmol/L and/or hemoglobin A1c (HbA1c) ≥ 6.5% (14). The HF diagnostic criteria were in accordance with Chinese Guidelines for the Diagnosis and Treatment of Heart Failure 2018 (15). Studies involving human participants were reviewed and approved by the Ethics Committee of Southern Medical University (The First People’s Hospital of Shunde). The approval number from the Ethics Committee is 20190525. The patients/participants provided their written informed consent to participate in this study.

### Data Collection

We collected baseline data from the patients at early hospital admission: (1) patient characteristics including age group (≤ 29 years, 30∼60 years, and ≥ 61 years), sex, and history of smoking and drinking; (2) clinical laboratory data included high-density lipoprotein (HDL), low-density lipoprotein (LDL), estimated glomerular filtration rate (eGFR), and aldehyde dehydrogenase 2 (*ALDH2*) gene; (3) cardiovascular conditions including blood pressure and history of coronary heart disease (CHD). We combined the results of published studies with actual clinical examination (16–20), and we used age group, sex, history of smoking and drinking, HDL, LDL, *ALDH2* and cardiovascular conditions as the predictor variables.

### Model Development and Validation

Least absolute shrinkage and selection operator (Lasso) logistic regression was conducted to select the optimal predictive factors. A model with excellent performance and the least number of independent variables was given when we adopted the lambda.1se. A multivariate logistic regression analysis was conducted to develop a prediction model by incorporating the variables selected in the Lasso model. The created model was tested in internal validation with the bootstrap resampling technique, in which regression models were fitted in 100 bootstrap replicates. The performance of the model was expressed as the following indicators:

1. A calibration plot was used to estimate the calibration of the final model and bootstrap validation. The difference between the average of observed outcomes and the average of predicted probabilities was reflected with the plot.
2. A *R*^2^ statistic was conducted to evaluate the goodness of fit test of the model. The closer the value to 1, the better the model fits the sample observations.
3. The area under curve (AUC) was used to evaluate the discrimination of the model. If the model can better differentiate among patients who did or did not have HF, the result of AUC was close to 1.
4. Brier score was used to measure the error between the probability of the category predicted by the model and the real value.

All the development and validation of model were analyzed with R version 4.1.2 (R Foundation for Statistical Computing, Vienna, Austria). The R code of the developed model is available in Supplementary Material 1. Our study was in accordance with the TRIPOD statement (Supplementary Material 2). Continuous data were expressed as median (25th, 75th) and categorical variables as frequencies and percentages. Some data were missing for all the risk factors except for age, sex, eGFR, and the *ALDH2* gene. We filled in missing values with the method of mean/mode completer.

## Results

### Baseline Characteristics of Patients

A total of 270 patients with DM were included in this analysis. Of the 270 patients, there were 188 patients diagnosed with DM-HF; 77.7% of the patients with DM-HF were ≥ 61 years, and 62.2% of the patients with DM were 30∼60 years. The median of LDL was 2.39 mmol/L (interquartile range 1.85∼3.15) in the DM-HF group and 1.52 mmol/L (interquartile range 0.98∼2.45) in the DM group; 28.7% of the patients with DM-HF had CHD, while only 6.1% of the patients with DM had this disease. There were significant differences in age group, HDL, LDL, eGFR, and CHD between the two groups. The baseline characteristics of patients in the two groups are summarized in Table 1.

**TABLE 1:** Baseline characteristics of patients included.

Variables	DM-HF	DM	*P*
Patients, *n*	188	82	
**Sex**			
Male, *n* (%)	111 (59.0)	45 (54.9)	0.524
**Age group, *n* (%)**			
≤ 29	2 (1.1)	10 (12.2)	
30∼60	40 (21.3)	51 (62.2)	
≥ 61	146 (77.7)	21 (25.6)	**< 0.01**
HDL, mmol/L	1.04 (0.87∼1.25)	1.33 (1.12∼1.72)	**< 0.01**
LDL, mmol/L	2.39 (1.85∼3.15)	1.52 (0.98∼2.45)	**< 0.01**
eGFR, ml/min	102.82 (76.02∼131.39)	62.64 (39.98∼84.24)	**< 0.01**
**ALDH2, *n* (%)**			
A	19 (10.1)	8 (9.8)	
B	73 (38.8)	28 (34.1)	
C	96 (51.1)	46 (56.1)	0.735
Smoking, *n* (%)	65 (34.6)	24 (29.3)	0.394
CHD, *n* (%)	54 (28.7)	5 (6.1)	**< 0.01**
Systolic pressure, mmHg	131 (117∼150)	127 (120∼135)	1.000
Diastolic pressure, mmHg	79 (70∼93)	81 (75∼92)	0.998
Drinking, *n* (%)	36 (19.1)	17 (20.7)	0.763

*DM, diabetes mellitus; HF, heart failure; CHD, coronary heart disease; HDL, high-density lipoprotein; LDL, low-density lipoprotein; ALDH2, aldehyde dehydrogenase 2; A, Lys504Lys; B, Glu504Lys; C, Glu504Glu. The bold P-values mean P < 0.05 and have a significant difference between groups.*

### Development of the Model

**FIGURE 1:**
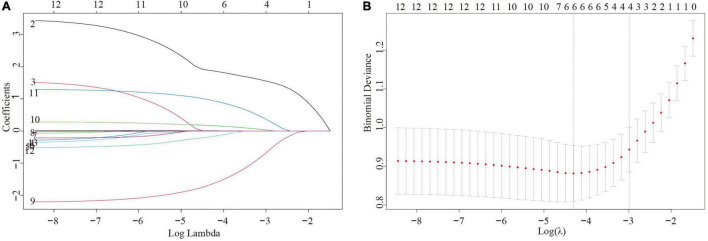
Variable selection using the least absolute shrinkage and selection operator (Lasso) logistic regression model. **(A)** Variable screening process using the control parameter log(λ) **(B)** The 50-fold cross-validation method.

Lasso regression was used to screen the four indicators involved in the establishment of the model (Figures 1A,B). The selected indicators were age, LDL, HDL, and CHD. The final multivariate regression model is shown in Table 2. Age ≥ 61 years old, LDL, and CHD were risk factors for DM-HF, with odds ratios (ORs) of 32.84 (95% CI: 6.74, 253.99), 1.33 (95% CI: 1.06, 1.72), and 3.94 (95% CI: 1.43, 13.43), respectively. HDL was a protective factor for DM-HF with an OR of 0.11 (95% CI: 0.04, 0.28).

**TABLE 2:** Final multivariate regression model of DM-HF.

Intercept	Multivariate regression		
	β	OR (95% CI)	*P*-value
Intercept	0.15	1.16(0.10∼9.18)	0.89
**Age group**			
≤ 29	1.00 (referent)		
30∼60	1.56	4.78(0.99∼36.26)	0.07
≥ 61	3.49	32.84(6.74∼253.99)	**< 0.001**
HDL	–2.21	0.11(0.04∼0.28)	**< 0.001**
LDL	0.29	1.33(1.06∼1.72)	**0.02**
**CHD**			
No	1.00 (referent)		
Yes	1.37	3.94(1.43∼13.43)	0.01

*OR, odds ratio; CI, confidence interval. The bold P-values mean P < 0.05 and have a significant difference between groups.*

### Performance of the Model and Internal Validation

**FIGURE 2:**
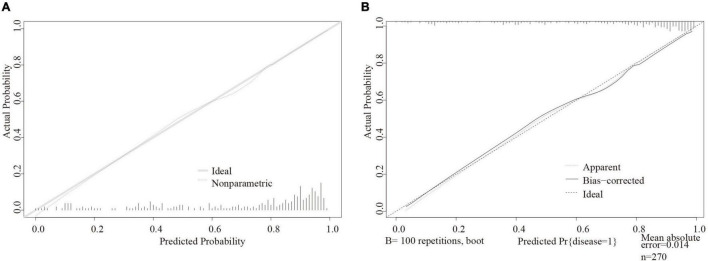
Calibration plot in the final regression nomogram and bootstrap validation. **(A)** Calibration curve for diabetes mellitus with heart failure in the development model. **(B)** Calibration curve for diabetes mellitus with heart failure in the internal validation.

We drew calibration curves to assess the degree of calibration of the risk prediction and internal validation of the DM-HF model (Figures 2A,B). As shown in Figure 2A, the x-axis represents the predicted risk for DM-HF, and the y-axis represents the actual probability of DM-HF. The dotted line represents the realized prediction power, and the solid line represents the prediction of an ideal model. The results showed a good consistency between the nomographic model and the ideal model. The calibration curve of the model also demonstrated a good agreement in the bootstrap validation cohort (Figure 2B).

The results in Table 3 show that the final logistic regression model has good discrimination for DM-HF (AUC = 0.863; 95% CI: 0.812∼0.913; *R*^2^ = 0.477; Brier-score = 0.128). The results of the internal validation indicated that there was negligible model optimism in bootstrap resampling (AUC = 0.845; *R*^2^ = 0.418; Brier score = 0.137).

**TABLE 3:** Model performances for final regression model and internal validation model.

	Final model	Internal validation
AUC-ROC	0.863(0.812∼0.913)	0.845
*R* ^2^	0.477	0.418
Brier-score	0.128	0.137
Slope	1.000	0.869

### Model Presentation

**FIGURE 3:**
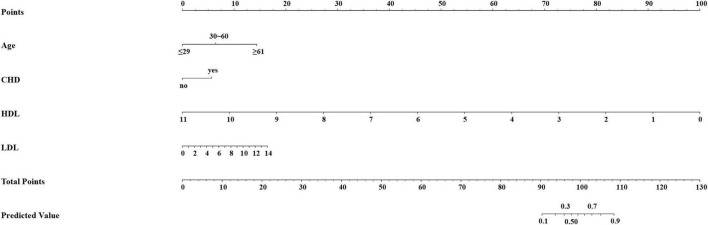
Prediction nomogram of diabetes mellitus with heart failure. The nomogram was developed with age, CHD, HDL, and LDL. CHD, coronary heart disease; HDL, high-density lipoprotein; LDL, low-density lipoprotein.

The final prediction model of DM-HF was displayed as a nomogram (Figure 3). We can score according to each variable, and the sum of all the variable scores is the total score. The total score on the DM-HF-predicted value axis represents the probability of DM-HF. The higher the score, the higher the risk that a patient with DM will develop HF. The web calculator can be linked through this URL: Nomogram For Diabetes Mellitus with Heart Failure (shinyapps.io).

## Discussion

In our study, a simplified model to predict the risk of DM-HF was constructed and successfully internally validated. The model showed good calibration and discrimination. We also provided the nomogram and web calculator to help one in calculating the risk.

Our study showed that age ≥ 61 years, LDL, and CHD were risk factors for DM-HF, and that high HDL was a protective factor for DM-HF. A multicenter study including 4,447 people concluded that more attention should be paid to elderly people to follow up on their risk of HF (21). In a systematic review and meta-analysis, the association between incident HF and 5-year increase in age (1.47; 1.25–1.73) was reported (19). In many studies, age was a risk factor for DM-HF (22, 23). Diabetic patients with low HDL level [3.62 (2.06–6.36)] have a higher risk of HF (24). Dyslipidemia is a risk factor for DM-HF. Lowering the level of LDL and increasing the level of HDL are engaging goals for reducing the risk of HF in patients with DM (25). The most common cause of HF is ischemic heart disease owing to impaired myocardial perfusion, while there are other common causes including DM and CHD (26). Murtaza et al. believed that long-term diabetes leads to structural and functional changes in the development and progression of HF, independent of myocardial ischemia or microvascular atherosclerotic disease (27).

Despite some published models can predict the risk of HF in patients with diabetes, our Lasso regression model still has its unique advantages. On the one hand, the population we included in our study was different from theirs. The data they used came from the Action to Control Cardiovascular Risk in Diabetes Study Group (ACCORD) trial, which was conducted in 77 centers across the United Stated and Canada (28, 29). All participants had established atherosclerotic coronary vascular disease or were 55–79 years of age with documented atherosclerosis, albuminuria, left ventricular hypertrophy, or two or more other cardiovascular risk factors, while our data came from Southern Medical University (The First People’s Hospital of Shunde), which is located in Chinese mainland and covers a catchment area of 3.2 million residents. On the other hand, we used a different statistical tool to construct the prediction model and visualization tools to show the model.

There are some limitations in our study. First, this is a retrospective study, and the results are inevitably biased. Second, the sample size in our research is very limited. In our subsequent research, we will enroll more patients for external verification. Third, some biomarkers had been well established to be associated with the risk of HF, including N-terminal pro B-type natriuretic peptide and soluble suppression of tumorigenesis-2 (30, 31). Furthermore, some other novel biomarkers, such as secreted frizzled-related protein 2 (SFRP2) (32, 33), trimethylamine N-oxide (TMAO) (34), and polyunsaturated fatty acids were also associated with the risk of HF (35). However, we did not include these biomarkers in our prediction model, as we aimed to provide metrics that can be easily extracted from clinical data. In conclusion, we conducted Lasso regression to screen variables and built a risk prediction model for diabetic HF. The model showed good discrimination and calibration in internal validation. A nomogram and a webpage calculator based on the model can make patients or doctors quickly calculate the risk of diabetic HF, which can help patients with diabetes reduce this risk better.

## Data Availability Statement

The datasets presented in this article are not readily available to uphold patient/participant privacy. Requests to access the datasets should be directed to the corresponding author.

## Ethics Statement

The studies involving human participants were reviewed and approved by the Southern Medical University (The First People’s Hospital of Shunde) of Ethics Committee. Written informed consent to participate in this study was provided by the participants’ legal guardian/next of kin.

## Author Contributions

HQ and WL contributed to the conception and design of the study. CW, PY, and WL organized the database. HQ performed the statistical analysis and wrote the manuscript. All authors contributed to manuscript revision and read and approved the submitted version.

## Conflict of Interest

The authors declare that the research was conducted in the absence of any commercial or financial relationships that could be construed as a potential conflict of interest.

## Publisher’s Note

All claims expressed in this article are solely those of the authors and do not necessarily represent those of their affiliated organizations, or those of the publisher, the editors and the reviewers. Any product that may be evaluated in this article, or claim that may be made by its manufacturer, is not guaranteed or endorsed by the publisher.
