# Supplementary material for: Development of Prediction Model to Estimate the Risk of Heart Failure in Diabetes Mellitus
Source: Front Cardiovasc Med. 2022 Jul 1;9:900267. doi: 10.3389/fcvm.2022.900267 (PMC9283704; doi:10.3389/fcvm.2022.900267)
Supplement: Supplementary file 1 [file Presentation_1.pdf]

R code of developing model

```
# ==== Predictor selection

# === define formula
Outcome <- "disease"
CandidateVariables                                                          <-
c("Age","Smoking","ALDH2","DP","SP","HDL","LDL","CHD","Gender","Drinking")

formula <- formula(paste(paste(Outcome,"~", collapse=" "),
                         paste(CandidateVariables, collapse=" + ")))
formula


# === fit full model
lrm_full <- lrm(formula, data = data )
lrm_full

model.full <- glm(formula, data=data,family=binomial)
model.full

summary(model.full)



# ====LASSO
tmp.y <- data$disease
tmp.x <- model.matrix(~.,data[CandidateVariables])
model.lasso <- glmnet(tmp.x, tmp.y, family="binomial", nlambda=50, alpha=1,
standardize=TRUE)
model.lasso

plot(model.lasso,xvar="lambda",label=TRUE)


# =====find the optimal model via cross-validation
cv.model <- cv.glmnet(tmp.x, tmp.y, family="binomial", nlambda=50, alpha=1,
standardize=TRUE)

plot(cv.model)

cv.model$lambda.min

coef(cv.model, s=cv.model$lambda.min)
```

```
# increase lambda for further shrinkage
cv.model$lambda.1se
coef(cv.model, s=cv.model$lambda.1se)


# ====== Fit the final model
FinalVariables <- c("Age", "CHD", "HDL", "LDL")

Formula <- formula(paste(paste(Outcome,"~", collapse=" "),
                         paste(FinalVariables, collapse=" + ")))
Formula

# ===glm way
model.final <- glm(Formula, data=data,family=binomial)

summary(model.final)

data$prob <- predict(model.final, data = data, type ="response")


exp(confint(model.final))

exp(coef(model.final))

model.final %>%
  tbl_regression(exponentiate =  TRUE)

# ====display the prediction model


subdt <- data[,c(Outcome,FinalVariables)]
subdt$CHD <- as.factor(subdt$CHD)
subdt$Age <- as.factor(subdt$Age)


dddt <- datadist(subdt)
options(datadist="dddt")

model.lrm <- lrm(Formula, data = subdt)

plot(nomogram(model.lrm, fun = plogis, lp = FALSE), xfrac=1.5, cex.axis=1.5)
```

```
plot(nomogram(model.lrm, fun = plogis, lp = FALSE), fun.at=c(0.1,seq(0.1,0.9,by=0.1),0.9 ))

# ==== internal validation


lrm.final <- lrm(formula = model.final$formula,   data = data,   x=TRUE,y=TRUE)

lrm.final

validate(lrm.final,method="boot",B=100)

plot(calibrate(lrm.final,method="boot",B=100))

#=== =C statistics


roc <- roc(data$disease, prob)
auc<- roc$auc

# ===AUC and 95% CI
auc
ci.auc(roc)
plot(roc)
summary(auc)

# === Brier score
data$disease <- as.factor(data$disease)
data$disease <- as.integer(data$disease)-1
brier <- mean((data$disease - prob)^2)
brier

#   === calibration
model.calibration <- glm(disease ~ lp, data = data, family = binomial)

# calibration intercept
model.calibration$coefficients[1]

# calibration slope
model.calibration$coefficients[2]

# ====Calibration slope and calibration curve


val.prob(p = prob,
```

```
y = data$disease,
logistic.cal=F,
# legendloc = FALSE,
statloc=F)
```
